# Supplementary material for: Major Allele Frequencies in CYP2C9 and CYP2C19 in Asian and European Populations: A Case Study to Disaggregate Data Among Large Racial Categories
Source: J Pers Med. 2025 Jun 27;15(7):274. doi: 10.3390/jpm15070274 (PMC12300455; doi:10.3390/jpm15070274)
Supplement: Supplementary file 1 [file jpm-15-00274-s001.zip › jpm-3698771-supplementary.pdf]

Supplementary Table S1

Population	Sample Size	Allele Frequency (%)					References
		CYP 2C9*2	CYP 2C9*3	CYP 2C9*5	CYP 2C9*8	CYP 2C9*11	
Overall European (Range)	3,040	9.9 – 15.7	5.3 – 9.8				
○ Croatian	429	14.7	7.6				1
○ Danish	276	12.1	5.3				2
○ Norwegian	309	9.9	6.5				2
○ Romanian	332	11.3	9.3				3
○ Serbian	500	11.7	8.1				4
○ Spanish	102	15.6	9.8				5
○ Spanish, Northern Italian	1,092	15.7	7.8				6
Overall Asian (Range)	41,197	0 – 4					
	45,916		0.5 – 18.9				
	4,130			0			
	800				1.8		
	5,962					0 – 0.05	
○ Chinese	7,840	0.3					7
	12,674		3.2				
	800			0	1.8		
	5,246					0.05	

○ Chinese	6,104	0	9.0				
○ Chinese	1,016	0					
○ Chinese	896		3.0				
○ Chinese	115		2.0				
○ Filipino	192	0.5	1.9				8,9
○ Filipino	99	0	1.0				
○ Hmong	433	0	18.9				10,11
○ Hmong	198	0	16.6				
○ Indian	5,862	4.0	9.0				12,13
○ Indian	1,868	4.0	8.0				
○ Japanese	4,554	0	2.0				7,8,12,14,15
○ Japanese	1,512	0					
	1,402		2.2				
○ Japanese	180	0	2.5				
	2,614			0			
○ Japanese	186	0	0.5				
○ Japanese	84	0	5.4				
○ Korean	7,120	0	4.0				7,8,12,14
	716			0		0	

o Korean	2,308	0	5.0				
o Korean	1,148	0	1.1				
o Korean	79	0	2.7				
o Vietnamese	314	0	2.2				[7,16]
o Vietnamese	100	0	3.5				

1. Celinscak Z, Zajc Petranovic M, Setinc M, et al. Pharmacogenetic distinction of the Croatian population from the European average. Croat Med J 2022;63(2):117-125. DOI: 10.3325/cmj.2022.63.117.
2. Pedersen RS, Brasch-Andersen C, Sim SC, et al. Linkage disequilibrium between the CYP2C19*17 allele and wildtype CYP2C8 and CYP2C9 alleles: identification of CYP2C haplotypes in healthy Nordic populations. Eur J Clin Pharmacol 2010;66(12):1199-205. DOI: 10.1007/s00228-010-0864-8.
3. Buzoianu AD, Trifa AP, Muresanu DF, Crisan S. Analysis of CYP2C9*2, CYP2C9*3 and VKORC1 -1639 G>A polymorphisms in a population from South-Eastern Europe. J Cell Mol Med 2012;16(12):2919-24. DOI: 10.1111/j.1582-4934.2012.01606.x.
4. Skadric I, Stojkovic O. Defining screening panel of functional variants of CYP1A1, CYP2C9, CYP2C19, CYP2D6, and CYP3A4 genes in Serbian population. Int J Legal Med 2020;134(2):433-439. DOI: 10.1007/s00414-019-02234-7.
5. Dorado P, Berecz R, Norberto MJ, Yasar U, Dahl ML, A LL. CYP2C9 genotypes and diclofenac metabolism in Spanish healthy volunteers. Eur J Clin Pharmacol 2003;59(3):221-5. DOI: 10.1007/s00228-003-0588-0.
6. Sanchez-Diz P, Estany-Gestal A, Aguirre C, et al. Prevalence of CYP2C9 polymorphisms in the south of Europe. Pharmacogenomics J 2009;9(5):306-10. DOI: 10.1038/tpj.2009.16.
7. Dorji PW, Tshering G, Na-Bangchang K. CYP2C9, CYP2C19, CYP2D6 and CYP3A5 polymorphisms in South-East and East Asian populations: A systematic review. J Clin Pharm Ther 2019;44(4):508-524. DOI: 10.1111/jcpt.12835.
8. Alrajeh K, AlAzzeh O, Roman Y. The frequency of major ABCG2, SLCO1B1 and CYP2C9 variants in Asian, Native Hawaiian and Pacific Islander women subgroups: implications for personalized statins dosing. Pharmacogenomics 2023;24(7):381-398. DOI: 10.2217/pgs-2023-0043.
9. Ustare LAT, Reyes KG, Lasac MAG, Brodit SE, Jr., Baclig MO. Single nucleotide polymorphisms on CYP2C9 gene among Filipinos and its association with post-operative pain relief via COX-2 inhibitors. Int J Mol Epidemiol Genet 2020;11(2):31-38. (https://www.ncbi.nlm.nih.gov/pubmed/33240461).
10. Sun B, Wen YF, Culhane-Pera KA, et al. Differences in Predicted Warfarin Dosing Requirements Between Hmong and East Asians Using Genotype-Based Dosing Algorithms. Pharmacotherapy 2021;41(3):265-276. DOI: 10.1002/phar.2487.
11. Wen YF, Culhane-Pera KA, Thyagarajan B, et al. Potential Clinical Relevance of Differences in Allele Frequencies Found within Very Important Pharmacogenes between Hmong and East Asian Populations. Pharmacotherapy 2020;40(2):142-152. DOI: 10.1002/phar.2360.

12. Gaikwad T, Ghosh K, Shetty S. VKORC1 and CYP2C9 genotype distribution in Asian countries. Thromb Res 2014;134(3):537-44. DOI: 10.1016/j.thromres.2014.05.028.
13. Jose R, Chandrasekaran A, Sam SS, et al. CYP2C9 and CYP2C19 genetic polymorphisms: frequencies in the south Indian population. Fundam Clin Pharmacol 2005;19(1):101-5. DOI: 10.1111/j.1472-8206.2004.00307.x.
14. Xie HG, Prasad HC, Kim RB, Stein CM. CYP2C9 allelic variants: ethnic distribution and functional significance. Adv Drug Deliv Rev 2002;54(10):1257-70. DOI: 10.1016/s0169-409x(02)00076-5.
15. Soga Y, Nishimura F, Ohtsuka Y, et al. CYP2C polymorphisms, phenytoin metabolism and gingival overgrowth in epileptic subjects. Life Sci 2004;74(7):827-34. DOI: 10.1016/j.lfs.2003.07.018.
16. Vu NP, Nguyen HTT, Tran NTB, et al. CYP2C19 genetic polymorphism in the Vietnamese population. Ann Hum Biol 2019;46(6):491-497. DOI: 10.1080/03014460.2019.1687750.

Supplementary Table S2

Population	Sample Size	Allele Frequency (%)			References
		CYP 2C19*2	CYP 2C19*3	CYP 2C19*17	
Overall European (Range)	2,394	11.1 – 16.3			
	1,309		0		
	2,034			19.6 – 25.5	
○ Croatian	429		0	23.9	1
○ Danish	276	15.0		20.1	2
○ German	237	15.2	0	25.5	3
○ Greek	283	13.1	0	19.6	4
○ Italian	360	11.1	0		5
○ Norwegian	309	15.2		22.0	2
○ Serbian	500	16.3		22.2	6
Overall Asian (Range)	22,186	20.5 – 53.8			
	21,192		0 – 15.6		
	5,085			0 – 17.9	
○ Chinese	11,358	29.1			7-11
	10,364		4.4		
	1,648			1.2	
○ Chinese	1,050	31.7	4.5	2.1	
○ Chinese	384	24.9	3.4	1.2	
○ Chinese	136	38.6	5.2		
○ Chinese	121	45.5	4.5		
○ Filipino	230	35.8-39.0	6.0 -8.5	0- 0.5	12,13
○ Filipino	52	39.0	6.0		
○ Hmong	198	42.2	0.3	0	14,15
○ Hmong	40	53.8	0	0	

○ Indian	1868	35.0	1.0		16-20
○ Indian	308	22.0	0		
○ Indian	206	40.2	0	17.9	
○ Indian	139	41.7	1.2		
○ Indian	102	35.2	0	10.2	
○ Japanese	2,388	30.0	11.3		7,11,12,21,22
○ Japanese	210	35.1	9.1	1.1	
○ Japanese	186	28.8	13.2		
○ Japanese	134	26.7	12.8		
○ Japanese	96	27.1	15.6		
○ Korean	1,296	28.3	8.6		7,12,23-25
	542			1.5	
○ Korean	377	28.3	7.6		
○ Korean	271	28.4	10.1	1.5	
○ Korean	104	33.8	8.2	1.3	
○ Korean	103	21.0	12.0		
○ Vietnamese	474	27.6	5.3		7,11,15,23
○ Vietnamese	165	26.4	4.9		
○ Vietnamese	100	20.5	2.5	1.0	
○ Vietnamese	90	23.6	13.9		

1. Celinscak Z, Zajc Petranovic M, Setinc M, et al. Pharmacogenetic distinction of the Croatian population from the European average. Croat Med J 2022;63(2):117-125. DOI: 10.3325/cmj.2022.63.117.
2. Pedersen RS, Brasch-Andersen C, Sim SC, et al. Linkage disequilibrium between the CYP2C19*17 allele and wildtype CYP2C8 and CYP2C9 alleles: identification of CYP2C haplotypes in healthy Nordic populations. Eur J Clin Pharmacol 2010;66(12):1199-205. DOI: 10.1007/s00228-010-0864-8.
3. Geisler T, Schaeffeler E, Dippon J, et al. CYP2C19 and nongenetic factors predict poor responsiveness to clopidogrel loading dose after coronary stent implantation. Pharmacogenomics 2008;9(9):1251-9. DOI: 10.2217/14622416.9.9.1251.
4. Ragia G, Arvanitidis KI, Tavridou A, Manolopoulos VG. Need for reassessment of reported CYP2C19 allele frequencies in various populations in view of CYP2C19*17 discovery: the case of Greece. Pharmacogenomics 2009;10(1):43-9. DOI: 10.2217/14622416.10.1.43.

5. Scordo MG, Caputi AP, D'Arrigo C, Fava G, Spina E. Allele and genotype frequencies of CYP2C9, CYP2C19 and CYP2D6 in an Italian population. Pharmacol Res 2004;50(2):195-200. DOI: 10.1016/j.phrs.2004.01.004.
6. Skadric I, Stojkovic O. Defining screening panel of functional variants of CYP1A1, CYP2C9, CYP2C19, CYP2D6, and CYP3A4 genes in Serbian population. Int J Legal Med 2020;134(2):433-439. DOI: 10.1007/s00414-019-02234-7.
7. Dorji PW, Tshering G, Na-Bangchang K. CYP2C9, CYP2C19, CYP2D6 and CYP3A5 polymorphisms in South-East and East Asian populations: A systematic review. J Clin Pharm Ther 2019;44(4):508-524. DOI: 10.1111/jcpt.12835.
8. Yang Z, Xie Y, Zhang D, et al. CYP2C19 gene polymorphism in Ningxia. Pharmacol Rep 2023;75(3):705-714. DOI: 10.1007/s43440-023-00473-5.
9. Chen L, Qin S, Xie J, et al. Genetic polymorphism analysis of CYP2C19 in Chinese Han populations from different geographic areas of mainland China. Pharmacogenomics 2008;9(6):691-702. DOI: 10.2217/14622416.9.6.691.
10. Zuo LJ, Guo T, Xia DY, Jia LH. Allele and genotype frequencies of CYP3A4, CYP2C19, and CYP2D6 in Han, Uighur, Hui, and Mongolian Chinese populations. Genet Test Mol Biomarkers 2012;16(2):102-8. DOI: 10.1089/gtmb.2011.0084.
11. Yamada S, Onda M, Kato S, et al. Genetic differences in CYP2C19 single nucleotide polymorphisms among four Asian populations. J Gastroenterol 2001;36(10):669-72. DOI: 10.1007/s005350170029.
12. Alrajeh KY, Roman YM. The frequency of major CYP2C19 genetic polymorphisms in women of Asian, Native Hawaiian and Pacific Islander subgroups. Per Med 2022;19(4):327-339. DOI: 10.2217/pme-2021-0175.
13. Goldstein JA, Ishizaki T, Chiba K, et al. Frequencies of the defective CYP2C19 alleles responsible for the mephenytoin poor metabolizer phenotype in various Oriental, Caucasian, Saudi Arabian and American black populations. Pharmacogenetics 1997;7(1):59-64. DOI: 10.1097/00008571-199702000-00008.
14. Wen YF, Culhane-Pera KA, Thyagarajan B, et al. Potential Clinical Relevance of Differences in Allele Frequencies Found within Very Important Pharmacogenes between Hmong and East Asian Populations. Pharmacotherapy 2020;40(2):142-152. DOI: 10.1002/phar.2360.
15. Vu NP, Nguyen HTT, Tran NTB, et al. CYP2C19 genetic polymorphism in the Vietnamese population. Ann Hum Biol 2019;46(6):491-497. DOI: 10.1080/03014460.2019.1687750.
16. Jose R, Chandrasekaran A, Sam SS, et al. CYP2C9 and CYP2C19 genetic polymorphisms: frequencies in the south Indian population. Fundam Clin Pharmacol 2005;19(1):101-5. DOI: 10.1111/j.1472-8206.2004.00307.x.
17. Gulati S, Yadav A, Kumar N, et al. Frequency distribution of high risk alleles of CYP2C19, CYP2E1, CYP3A4 genes in Haryana population. Environ Toxicol Pharmacol 2014;37(3):1186-93. DOI: 10.1016/j.etap.2014.03.013.
18. Anichavezhi D, Chakradhara Rao US, Shewade DG, Krishnamoorthy R, Adithan C. Distribution of CYP2C19*17 allele and genotypes in an Indian population. J Clin Pharm Ther 2012;37(3):313-8. DOI: 10.1111/j.1365-2710.2011.01294.x.
19. Ghodke Y, Joshi K, Arya Y, et al. Genetic polymorphism of CYP2C19 in Maharashtrian population. Eur J Epidemiol 2007;22(12):907-15. DOI: 10.1007/s10654-007-9196-0.
20. Shalia KK, Shah VK, Pawar P, Divekar SS, Payannavar S. Polymorphisms of MDR1, CYP2C19 and P2Y12 genes in Indian population: effects on clopidogrel response. Indian Heart J 2013;65(2):158-67. DOI: 10.1016/j.ihj.2013.02.012.
21. Kubota T, Chiba K, Ishizaki T. Genotyping of S-mephenytoin 4'-hydroxylation in an extended Japanese population. Clin Pharmacol Ther 1996;60(6):661-6. DOI: 10.1016/S0009-9236(96)90214-3.

22. Fukushima-Uesaka H, Saito Y, Maekawa K, et al. Genetic variations and haplotypes of CYP2C19 in a Japanese population. Drug Metab Pharmacokinet 2005;20(4):300-7. DOI: 10.2133/dmpk.20.300.
23. Lee SS, Lee SJ, Gwak J, et al. Comparisons of CYP2C19 genetic polymorphisms between Korean and Vietnamese populations. Ther Drug Monit 2007;29(4):455-9. DOI: 10.1097/FTD.0b013e31811f383c.
24. Kim KA, Song WK, Kim KR, Park JY. Assessment of CYP2C19 genetic polymorphisms in a Korean population using a simultaneous multiplex pyrosequencing method to simultaneously detect the CYP2C19*2, CYP2C19*3, and CYP2C19*17 alleles. J Clin Pharm Ther 2010;35(6):697-703. DOI: 10.1111/j.1365-2710.2009.01069.x.
25. Roh HK, Dahl ML, Tybring G, Yamada H, Cha YN, Bertilsson L. CYP2C19 genotype and phenotype determined by omeprazole in a Korean population. Pharmacogenetics 1996;6(6):547-51. DOI: 10.1097/00008571-199612000-00008.
